# Life skills, self-efficacy, life satisfaction and health literacy evolution from primary school to middle school: a 3-year longitudinal interventional study of the Explo’Santé cohort

**DOI:** 10.3389/fpubh.2026.1720265

**Published:** 2026-01-27

**Authors:** Corélie Salque, Adeline Darlington-Bernard, Florence Carrouel, Emily Darlington

**Affiliations:** Laboratory Health Systemic Process (P2S) UR4129, University Claude Bernard Lyon 1, University of Lyon, Lyon, France

**Keywords:** evaluation, health education, health literacy, health promotion, life satisfaction, life skills, self-efficacy

## Abstract

**Introduction:**

Childhood and adolescence are decisive life stages during which social and health inequalities emerge and widen. Schools represent a privileged setting for health promotion, particularly through the development of psychosocial resources such as life skills (LS), self-efficacy (SE), life satisfaction (LSa), and health literacy (HL). The Explo’Santé program is a French school-based health promotion intervention that combines structured health education sessions with supportive environments.

**Methods:**

This longitudinal cohort study followed 744 pupils from 4th grade in primary school to the first year of middle school. LS, SE, LSa, and HL were assessed at six time points across three annual intervention cycles, each including ten weekly school-based health education sessions.

**Results:**

Linear mixed models showed short-term gains in LS, SE, and LSa during primary school, followed by a marked decline after the transition to middle school, whereas HL increased steadily throughout the 3 years. Girls consistently scored higher than boys but also showed sharper decreases once in 6th grade. Territorial disparities were observed, with some districts showing strong improvements while others consistently lagged.

**Discussion:**

These findings suggest that the Explo’Santé program supports the development of key psychosocial competencies, but that the transition to middle school represents a critical turning point which challenges their sustainability. By highlighting gendered vulnerabilities and contextual inequalities, this work contributes to understanding how schoolbased health promotion can foster more equitable developmental trajectories.

## 1 Introduction

Childhood and adolescence are decisive developmental periods during which social, emotional, and health inequalities emerge and often widen (1, 2). From the first years of primary school, disparities can already be observed in well-being, coping resources, and access to health-promoting environments. Recent data confirm these early gaps: in Europe, about 13% of schoolchildren aged 6–11 have a “probable” mental health problem (3). In the Organisation for Economic Cooperation and Development (OECD)countries, nearly 18% adolescents reported low life satisfaction in 2022, compared to 12% in 2015 (4). These early differences tend to persist in adolescence, shaping both educational attainment and long-term health trajectories. Evidence shows, for instance, that health-related behaviors established before age 14 strongly predict risks of obesity, cardiovascular disease, and mental health disorders later in life (5).

This interdependence between education and health has led to a growing recognition that schools are not only places of academic instruction but also privileged settings for reducing inequalities and fostering holistic development (6). Since almost all children spend a large part of their daily lives in school, the educational environment offers a unique and universal entry point for promoting health in an equitable and sustainable way (7). With over 95% of children in Europe enrolled in school (8), and with two-thirds identifying school pressure as a major source of stress (9), schools represent both a challenge and an opportunity for mental and physical health promotion.

In this perspective, health education is increasingly conceived not as the mere transmission of knowledge about risk behaviors or healthy lifestyles. It is understood as the development of psychosocial and cognitive resources that empower children to navigate daily challenges, transitions and uncertainties. The World Health Organization defines life skills (LS) as “abilities for adaptive and positive behavior that enable individuals to deal effectively with the demands and challenges of everyday life.” Closely related constructs further highlight their multidimensional nature Firstly, self-efficacy (SE) was conceptualized in Bandura’s social cognitive theory as the belief in one’s ability to organize and execute courses of action required to manage prospective situations (10). In the school context, SE is defined as a transversal perception of one’s capacity to understand and respond to the expectations of the school environment across disciplines (11). Secondly, health literacy (HL) is defined as “people’s knowledge, motivation and competencies to access, understand, appraise, and apply health information in order to make judgments and take decisions in everyday life” (12). Finally, life satisfaction (LSa) is framed as the cognitive evaluation of one’s overall quality of life (13). Large-scale international surveys such as the Health Behavior in School-aged Children (HBSC) and the Programme for International Student Assessment (PISA) have repeatedly shown that these psychosocial indicators are socially patterned and linked both to educational outcomes and to long-term health inequalities (14, 15).

Together, these psychosocial indicators —interlinked and mutually reinforcing—have been shown to support both academic success and health outcomes (16–18). This positions them at the crossroads of education and public health and underlines their potential as levers for reducing social and health inequalities from an early age (1, 19).

Nevertheless, evaluating the effectiveness of school-based health promotion programs is complex. Interventions are implemented in diverse contexts, shaped by school resources, socioeconomic environments, and local dynamics that can strongly influence outcomes (20, 21). Program impacts may also vary over time, particularly as children move up from primary to middle school, a period marked by profound developmental, social, and institutional changes (22, 23). This primary–secondary school transition is understood as a complex and multidimensional process involving shifts in relationships, identity, school environments, and academic expectations, often overlapping with broader developmental changes (24). This raises key methodological challenges: assessing not only short-term effects but also long-term developmental trajectories, while accounting for both individual factors (e.g., gender, socioeconomic context) and contextual ones (e.g., school, district) (6, 21). Despite a growing literature on school health promotion, relatively few studies have followed large cohorts of children longitudinally, with designs capable of disentangling program effects from contextual influences across critical educational transitions (25–27).

The Explo’Santé program was developed in this perspective, as a universal school-based health education initiative grounded in LS development. Its pedagogical framework integrates health promotion principles with educational objectives, aiming to strengthen pupils’ psychosocial resources and to support more equitable educational pathways (28). In doing so, it aligns with the WHO Health Promoting Schools framework, which emphasizes the integration of health into the core mission, culture, and environment of schools (6). By embedding health promotion within the ordinary functioning of schools, the program seeks not only to improve individual skills but also create learning environments that are more supportive, inclusive, and conducive to health and learning (6, 29). In the French education system, health education is not delivered as a standalone subject. It is proposed within the scope of the *Parcours Éducatif de Santé* (Health Education Pathway (HEP)) (30). This scheme covers screening and vaccination campaigns, and prevention and health promotion intervention, including health education interventions, for all pupils from nursery school to the end of high school. Its objective is to accompany pupils to develop their knowledge and competences, to make their own choices and to behave responsibly towards themselves and others (31). Although its design is specific to each school and defined by the teachers, the school nurse and the school district doctor. In primary school, health is addressed either through cross-disciplinary competencies and prevention-related themes integrated across subjects. At the beginning of lower secondary school (Grade 6), this remains the case. In addition, the activities of the school board includes the *Comité d’Éducation à la Santé et la Citoyenneté* (School Health and Citizenship Committee (SHCC)). It gathers representatives from the school community to implement health-related projects and interventions. Against this backdrop, Explo’Santé falls within the scope of both the French HEP and SHCC. It is proposed by the French League against Cancer as a structured LS-based program. It is delivered within regular school hours, either within standard lessons in primary school, or, in 6th grade, during class meetings or slots freed from other subjects. It offers a coherent and operational framework that extends and strengthens the existing health education mandate. Evaluating such a program offers a valuable opportunity to explore the potential of school health promotion to mitigate inequalities, while at the same time fostering psychosocial development and encouraging healthier pathways across educational transitions (5).

This study aims to evaluate the Explo’Santé intervention through a three-year longitudinal follow-up of pupils from primary to middle school. Specifically, it seeks to (1) describe the overall trajectories of LS, SE, LSa and HL across the six measurement points; (2) examine whether these trajectories differ by gender and district; and (3) assess individual and contextual variability using mixed-effects models. Together, these aims provide a comprehensive understanding of how pupils’ psychosocial resources evolve over time and how the program’s effects may vary across social and school contexts. More broadly, it addresses the challenges and contributions of evaluating school-based health education initiatives within a health promotion framework, aiming to advance both scientific knowledge and practical perspectives in this evolving field.

## 2 Materials and methods

### 2.1 Study design

This study was designed as a longitudinal cohort study (28). The protocol was approved by the University of Lyon’s ethics committee (France) on March 1, 2023 (n°2023-01-12-004). This manuscript follows the guidelines for reporting observational epidemiological data of the STROBE checklist (32) (Supplementary Table 1).

### 2.2 Setting

The study was conducted in six administrative districts of France (Districts A–F). A cohort of 744 pupils was recruited during their 4th grade year between March 1 and March 13, 2023. At baseline, the 4th grade pupils were distributed across 60 classes in 43 primary schools. They were subsequently followed as they progressed into 59 classes in 5th grade and 32 classes in 6th grade, within 8 middle schools.

The follow-up period extended over three academic years (2022–2025). Data were collected at six time points, at the beginning and end of each school year, using anonymous self-administered questionnaires completed in the classroom under teacher supervision (see Supplementary Figure 1).

### 2.3 Participants

Pupils were eligible for inclusion if they met the following criteria: (i) being enrolled in 4th grade during the first year of the study (2022–2023), (ii) attending an institution participating in the Explo’Santé program over the three consecutive school years (2022–2023, 2023–2024, and 2024–2025), (iii) being fluent in French, (iv) having written parental consent, and (v) providing oral assent prior to completing the questionnaires.

### 2.4 Intervention

Explo’Santé is a three-year school-based health promotion program developed by the French League Against Cancer. It followed pupils from grade 4 to grade 6 and combined two complementary components: (i) classroom-based health education focused on life skills, delivered through 10 participatory sessions per year co-facilitated by prevention officers and teachers, and (ii) the creation of healthy school environments (e.g., smoke-free spaces, improved playgrounds, strengthened school climate). Pedagogical methods included role-plays, collaborative activities, and “take-home booklets” to involve families. Teachers received prior training, and local stakeholders were mobilized to ensure sustainability.

### 2.5 Variables

The primary outcomes were the pupils’ scores in LS, SE, LSa and HL. The main exposure of interest was participation in the Explo’Santé program. Predictors included time (distinguishing between primary and middle school to account for the transition effect), pupils’ gender, and the social position index (SSI) of their institution, ranging from 45 to 185, as a proxy for socioeconomic background. District and school-level context (class and institution), as well as individual pupil trajectories, were considered potential effect modifiers, as these hierarchical levels may influence the magnitude of program effects across contexts.

### 2.6 Data measurement

Data were collected anonymously at six time points (Supplementary Figure 1) by three researchers using electronic tablets. The questionnaires used were composed of four validated scales. Firstly, the LS scale (30 questions, 5-item Likert scale), was composed of a cognitive LS scale (33), an emotional LS scale (34), and a social LS scale (35). They were translated in French from the Assessment of Children’s Emotion Skills (ACES) (36) and the Resolving Conflicts, Helping Others, Managing Emotions sections of the Life Skills Transfer Survey (37). Secondly, the original SE perception at school scale (17 questions, 6-item Likert scale) includes a school SE in general scale, an SE in French scale, and an SE in Maths scale (11). Thirdly, the LSa scale (30 questions, 4-item Likert scale; 67) comprises 5 dimensions: family, living environment, friends, school, and self. It is a French version of Huebner’s Multidimensional Students’ Life Satisfaction Scale (MSLSS) (38). Finally, the HL scale (10 questions, 4-item Likert scale) (39) is a translated version of the Health literacy for School-Aged Children scale (HLSAC) (40), validated for students from 5th grade (41). These four instruments were validated with French school-aged populations, with a reliability estimated satisfactory to excellent (LS (*α* = 0.74–0.90), SE (*α* = 0.74–0.92), LSa (*α* = 0.70–0.90), HL (*α* = 0.82–0.89)) (42). These questionnaires were administered to all students across time points to ensure comparability.

Each pupil was assigned a unique identification code combining district and primary school identifiers, which enabled longitudinal follow-up. Gender was self-reported at the beginning of each questionnaire. Contextual data on institutions’ Socioeconomic Status Index (SSI) were extracted from the INSEE national database (43). Data entry was automated through electronic devices, minimizing transcription errors.

### 2.7 Bias

Efforts were made to minimize potential sources of bias. Firstly, data collection was standardized across schools and time points using validated self-administered questionnaires, which reduced interviewer bias but may still be subject to self-report bias. Secondly, attrition across time points was addressed through both complete-case and whole-data analyses. Thirdly, hierarchical mixed models accounted for clustering at class and institutional levels.

### 2.8 Study size

The study size was determined *a priori* using Cochran’s formula, assuming a 50% prevalence of satisfactory LS to maximize statistical variance in the absence of prior data. With a 95% confidence level and a ± 5% margin of error, the minimum required sample size was estimated at 385 pupils. In practice, 744 pupils aged 8–10 at baseline (44) participated in the study, which provided sufficient power for the planned analyses. Among them, 254 pupils completed all questionnaires across the follow-up period; this complete-case subset was used exclusively for sensitivity analyses to assess the robustness of the findings.

### 2.9 Quantitative variables

Quantitative variables were analyzed as continuous measures to preserve statistical power and variability. Scores from the four scales and their subscales were normalized to a uniform range of 0–100 to facilitate comparability across instruments. The SSI was used as a continuous proxy for socioeconomic background; no arbitrary cut-offs were applied to avoid loss of information. Time was operationalized both as repeated measures at pupil level and at school stage (primary vs. middle school) to capture potential contextual effects. No additional categorization or grouping was performed beyond these theoretically justified distinctions.

### 2.10 Statistical methods

All statistical analyses were performed in RStudio. Descriptives were first used to summarize participant characteristics by time point, gender, and district. Repeated-measures ANOVA were conducted to examine overall time effects on LS, SE, LSa, and HL scores, followed by Bonferroni-adjusted post-hoc pairwise comparisons. To account for individual and contextual variability, mixed-effects linear regression models with random intercepts for pupils were performed. Models sequentially included gender, district, and school level, with and without adjustment for the Socioeconomic Status Index (SSI), to disentangle contextual from socioeconomic effects. Subscale analyses were conducted for LS (cognitive, emotional, social), LSa (family, school, friends, living environment, self), and SE (general, French, mathematics). Missing data were imputed by the mean at scale level for mixed models on the full dataset. As loss to follow-up could not be avoided in this longitudinal design, sensitivity analyses were performed by comparing complete-case (*n* = 254) and full datasets (*n* = 744) to assess robustness of findings. Statistical significance was set at *p* < 0.05, with borderline significances noted for 0.05 ≤ *p* < 0.10.

### 2.11 Ethics

The protocol was approved by the University of Lyon’s ethics committee (France) on March 1, 2023 (n°2023-01-12-004). CNIL reference: 2226244 v 0.

Anonymity was ensured during data collection, data handling and data analysis. No risk was identified from participating in the study.

## 3 Results

### 3.1 Participants

**Figure 1:**
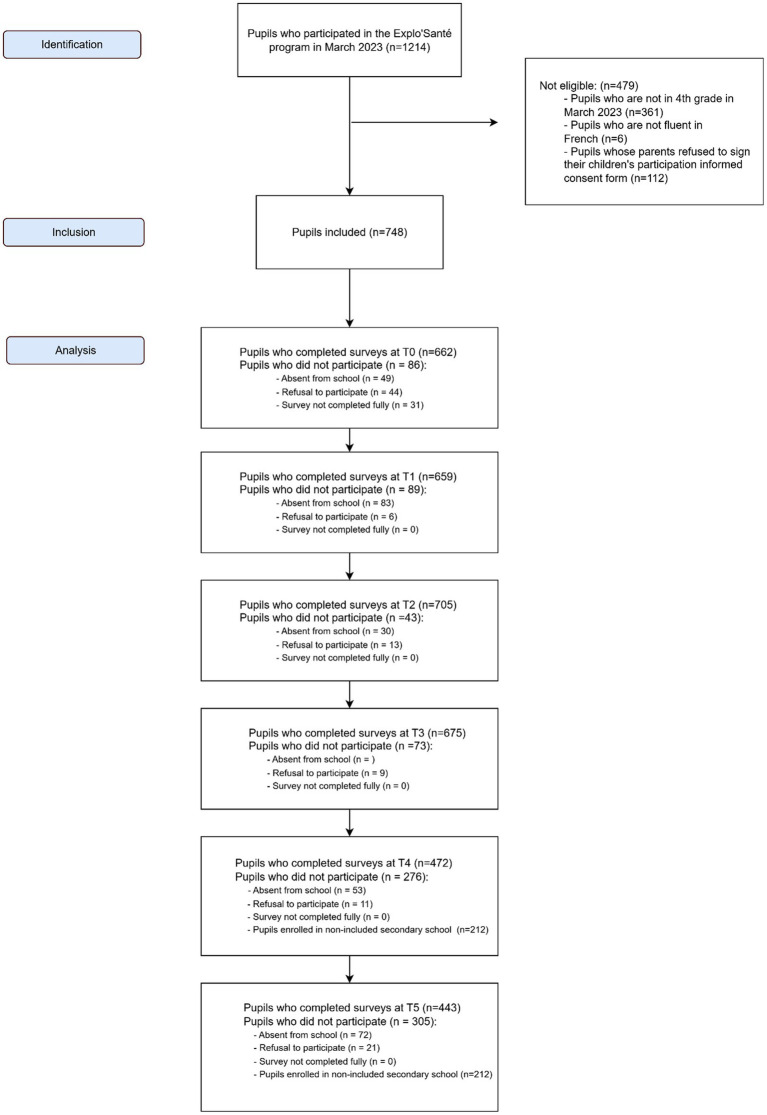
Flowchart of the study.

At baseline, 662 pupils completed the questionnaires. Participation remained steady at T1 (659) and slightly increased at T2 (705), before declining to 472 at T4, and 443 at T5. Attrition was mainly due to pupil absence, school transfer, or withdrawal of consent. Despite this, 726 pupils contributed repeated measures, enabling longitudinal analyses. Flow diagram is provided in Figure 1.

Table 1 summarizes the distribution of participants by gender and district at each time point, while the gender distribution and mean SSI across the six districts are addressed in Supplementary Table 2. Gender distribution was relatively balanced across time points, with a slight predominance of girls (52–55%). District representation varied: District A consistently accounted for about one quarter of participants (~25%), whereas Districts B and C contributed less than 10% each. Socioeconomic context, assessed using the SSI, showed moderate variability across schools, with higher average scores in District A and lower ones in District E.

**Table 1:** Population distribution according to gender and district.

Time	T0	T1	T2	T3	T4	T5	All times
Variables	*N* (%)	*N* (%)	*N* (%)	*N* (%)	*N* (%)	*N* (%)	*N* (%)
Overall	662 (100)	659 (100)	705 (100)	675 (100)	472 (100)	443 (100)	744 (100)
Missing	82 (11.02)	85 (11.42)	39 (5.24)	69 (9.27)	272 (36.56)	301 (40.46)	–
Gender							
Girls	344 (51.96)	347 (52.66)	368 (52.20)	361 (53.48)	259 (54.87)	242 (54.63)	390 (52.42)
Boys	318 (48.04)	312 (47.34)	337 (47.80)	314 (46.52)	213 (45.13)	201 (45.37)	354 (47.58)
District							
A	159 (24.02)	165 (25.04)	177 (25.11)	168 (24.89)	144 (30.51)	127 (28.67)	186 (25.00)
B	64 (9.67)	53 (8.04)	65 (9.22)	68 (10.07)	48 (10.17)	47 (10.61)	69 (9.14)
C	52 (7.85)	53 (8.04)	65 (9.22)	67 (9.93)	52 (11.02)	45 (10.16)	66 (8.87)
D	169 (25.53)	171 (25.95)	175 (24.82)	139 (20.59)	77 (16.31)	83 (18.74)	179 (24.06)
E	127 (19.18)	126 (19.12)	130 (18.44)	141 (20.89)	84 (17.80)	79 (17.83)	150 (20.16)
F	91 (13.75)	91 (13.81)	93 (13.19)	92 (13.63)	67 (14.19)	62 (14.00)	95 (12.77)

### 3.2 Life skills, self efficacy, life satisfaction and health literacy scores evolution

#### 3.2.1 Overall score evolution

**Figure 2:**
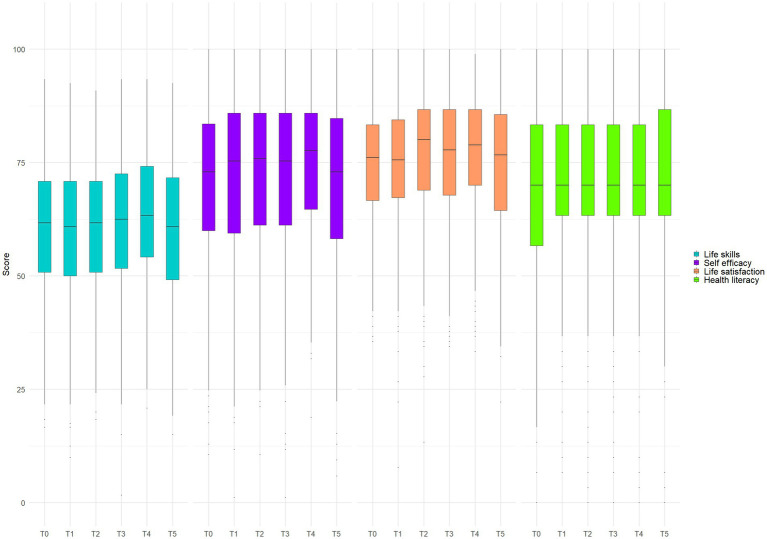
Boxplots of the life skills, self efficacy, life satisfaction, health literacy scores evolution between T0 and T5.

Descriptive statistics for all four scales at each measurement point (mean, n) for the full sample are presented in Supplementary Table 3. As shown in Figure 2 and Table 2, LS, SE, and LSa scores increased significantly from T0 to T4 before declining at T5. *Post-hoc* tests confirmed significant improvements at T4 compared with earlier time points, followed by significant decreases at T5. HL displayed a different trajectory, with steady but modest improvements from baseline to T3 and T5.

**Table 2:** Life skills, self efficacy, life satisfaction, and health literacy scores evolution with time: ANOVA and pairwise *post-hoc* tests.

Outcome	Time comparison^a^	Pairwise comparisons^b^ (β, p)	Overall time effect^c^
Life skills	T4 vs. T0	2.76, *p* < 0.001	*p* < 0.001
	T4 vs. T1	3.38, *p* < 0.001	
	T4 vs. T2	2.56, *p* < 0.001	
	T4 vs. T3	2.17, *p* = 0.010	
	T5 vs. T4	−3.44, p < 0.001	
Self-efficacy	T2 vs. T0	2.06, *p* = 0.014	*p* < 0.001
	T3 vs. T0	2.12, *p* = 0.013	
	T4 vs. T0	3.76, *p* < 0.001	
	T4 vs. T1	2.70, *p* = 0.002	
	T5 vs. T2	−2.35, *p* = 0.015	
	T5 vs. T3	−2.41, *p* = 0.012	
	T5 vs. T4	−4.04, *p* < 0.001	
Life satisfaction	T2 vs. T0	1.97, *p* = 0.002	*p* < 0.001
	T2 vs. T1	2.03, *p* < 0.001	
	T4 vs. T0	1.72, *p* = 0.041	
	T4 vs. T1	1.78, *p* = 0.029	
	T5 vs. T2	−3.57, *p* < 0.001	
	T5 vs. T3	−2.17, *p* = 0.003	
	T5 vs. T4	−3.32, *p* < 0.001	
Health literacy	T3 vs. T0	2.31, *p* = 0.032	*p* = 0.004
	T5 vs. T0	2.71, *p* = 0.021	

^a^Only significant results are presented. ^b^Bonferroni-adjusted test. ^c^Repeated-measures ANOVA test. Green indicates positive regression coefficients (β > 0), while yellow indicates negative coefficients (β < 0).

#### 3.2.2 Scores evolution according to gender

**Figure 3:**
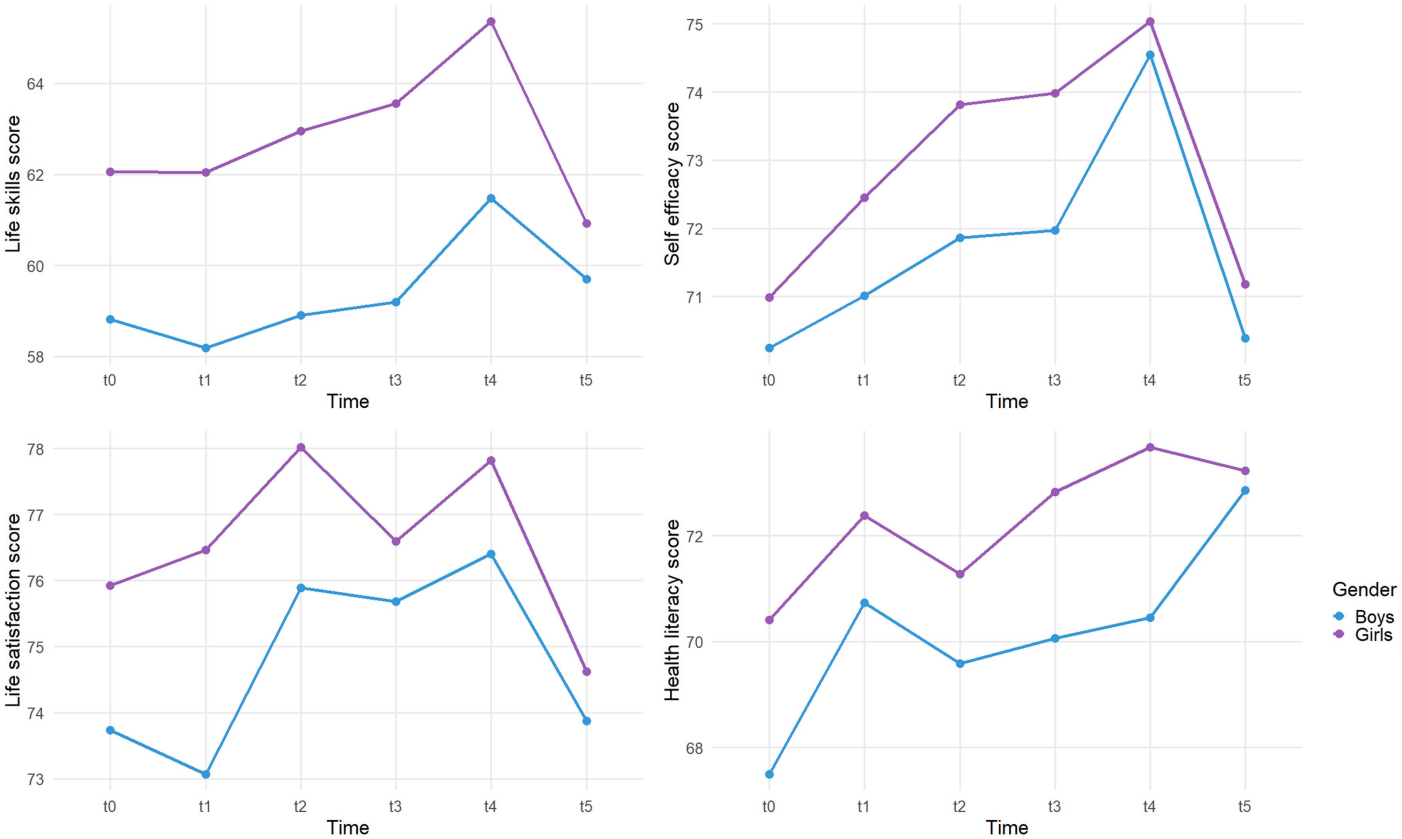
Life skills, self efficacy, life satisfaction, health literacy mean scores evolution between T0 and T5 according to gender.

Figure 3 illustrates LS, SE, LSa, and HL scores evolution by gender, with corresponding means and standard deviations reported in Supplementary Table 3. Across all outcomes and time points, girls tended to score higher than boys, although these differences gradually narrowed by T5. For LS, SE, and LSa, all genders showed an overall increase from T0 to T4 (e.g., +2.7, +4.3, +2.7 for boys, and +3.3, +4.0, +1.9 for girls, on average), marked by gains between T1–T2 and T3–T4, followed by a decline from T4 to T5 (e.g., −1.8, −4.1, −2.5 for boys, and −4.5, −3.8, −3.2 for girls, on average). Boys experienced an initial decrease in LS and LSa between T0 and T1, while all genders declined between T2 and T3. For HL, all genders peaked at T1, dropped between T1 and T2, and then rose steadily up to T4. At T4–T5, girls showed a slight decrease in HL, whereas boys continued to improve. By T5, girls had lost an average of 1.2 points in LS and 1.5 points in LSa compared to T0, while boys still exhibited a net increase from baseline across all outcomes.

#### 3.2.3 Scores evolution according to districts

**Figure 4:**
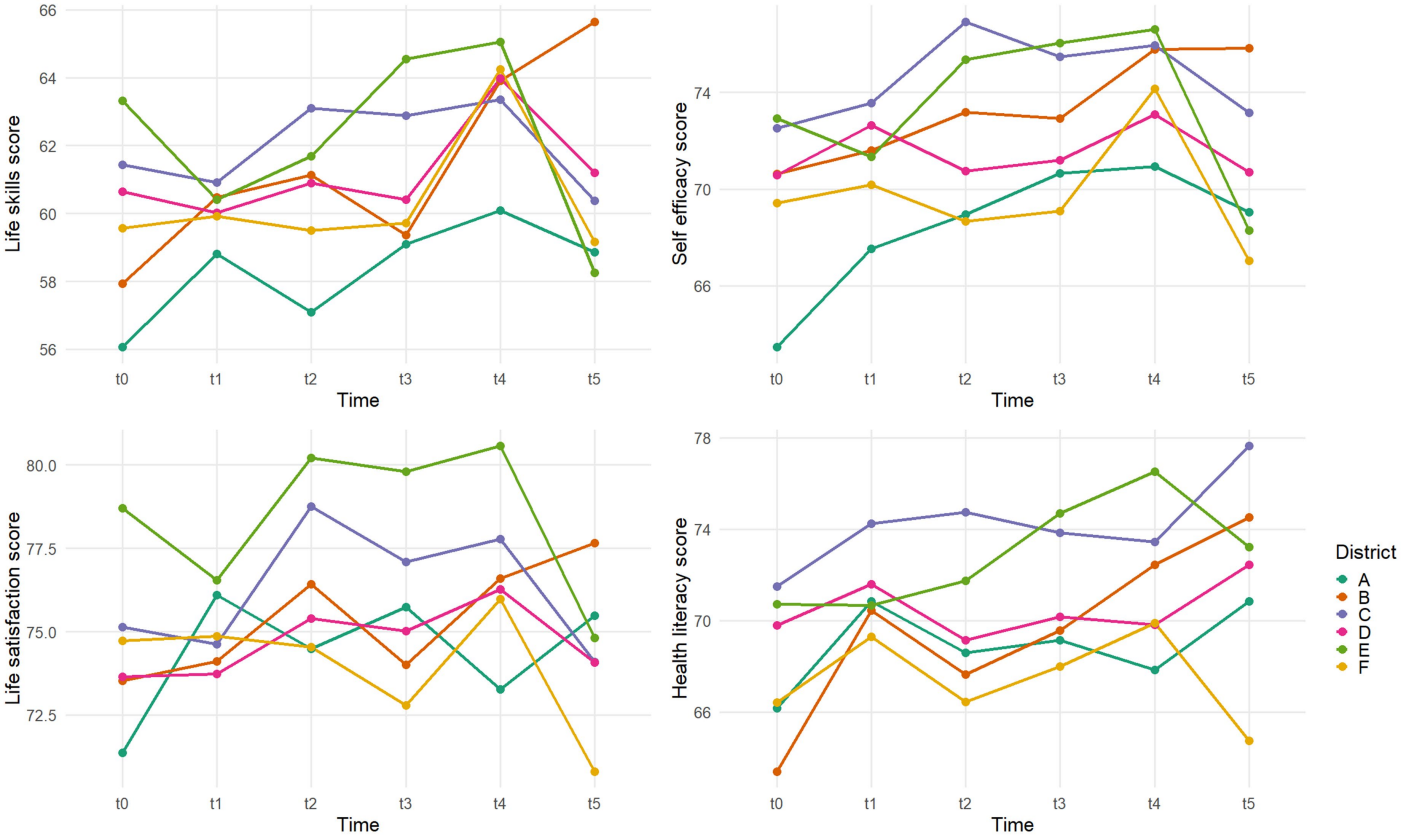
Life skills, self efficacy, life satisfaction, health literacy mean scores evolution between T0 and T5 according to district.

District effects are shown in Figure 4 and Supplementary Table 4. Districts A and E started with the highest scores and improved up to T4, both experienced steep declines at T5. District E, which had the highest LS scores at baseline (63.3), ended with the lowest LS scores at T5 (58.2). By contrast, District C, which started from relatively low baseline scores, showed the most favorable trajectory, gaining +7.7 in LS, +5.2 in SE, +4.2 in LSa, and +11.1 in HL, despite temporary declines in LS, SE, and LSa between T2–T3, and in HL from T0 to T5, and was the only district to increase LS and SE between T4 and T5. District B also showed overall improvement, despite very low baseline scores. In contrast, District F declined steadily after T4 and was the only district to finish below baseline across all outcomes.

### 3.3 Contextual and individual factors impacting life skills, self efficacy, life satisfaction and health literacy scores evolution

#### 3.3.1 Gender effect on life skills, self efficacy, life satisfaction and health literacy scores over time

Girls scored higher than boys on several outcomes (LS: +3.69, *p* < 0.001; LSa: +2.13, *p* < 0.01; HL: +2.09, *p* < 0.05; Table 3), which confirms the previous results. After accounting for SSI, the difference in HL was no longer significant, suggesting that SSI partly explained this gap.

**Table 3:** Life skills, self efficacy, life satisfaction, and health literacy scores evolution according to gender, district, and school level, with or without the socio-economic status index (SSI): Linear mixed models results.

Socioeconomic status index	Life skills	Self efficacy		Life satisfaction		Health literacy		
	NI	0.14	NI	*0.32*	NI	0.21	NI	0.44
Gender^a^	**3.69**	**3.59**	1.53	1.24	**2.13**	**2.12**	**2.09**	*1.92*
District^b^								
B	**−4.32**	*−4.46*	**−7.05**	**−7.77**	*−3.07*	**−4.32**	**−5.73**	**−6.71**
C	−0.80	−0.84	−1.76	−1.79	−1.31	−1.50	**−5.01**	**−5.46**
D	−1.38	−1.30	−2.80	*−3.81*	−2.17	**−3.77**	*−3.31*	**−4.57**
E	0.43	0.78	−0.63	−2.59	*2.46*	−0.45	−0.94	−3.30
F	−1.55	−1.43	**−3.71**	**−5.08**	−1.80	**−3.59**	**−6.09**	**−7.39**
Time in primary school	0.12	0.14	*0.08*	*0.32*	0.23	0.21	0.37	0.44
Time in middle school	**−2.70**	**−2.68**	**0.03**	*−1.70*	**−2.45**	**−2.24**	1.28	1.06

Significant results (*p* < 0.05) are in bold and marginally significant results (*p* < 0.1) are in italics. Reference: boys of District A at T0. NI: not included. The color scale reflects the sign and magnitude of the β coefficients: red denotes negative effects, green positive effects, and yellow coefficients close to zero.

At the subscale level, the LS girls’ advantage was mainly due to cognitive LS (+3.13, *p* < 0.001) and social LS (+5.63, *p* < 0.001) (Table 4).

**Table 4:** Cognitive, emotional and social life skills scores evolution according to gender, district, and school level: Linear mixed models results.

Predictor	**Cognitive life skills**	**Emotional life skills**	**Social life skills**
Gender	**3**.**13**	0.98	**5**.**63**
District^b^			
B	−2.85	−2.82	**−6**.**35**
C	−0.95	−1.28	−0.38
D	0.40	−0.08	−3.68
E	**3**.**61**	−1.88	−1.29
F	0.44	−1.23	−3.99
Time in primary school	−0.26	0.32	0.29
Time in middle school	**−3**.**32**	−0.54	**−2**.**72**

Significant results (*p* < 0.05) are in bold and marginally significant results (*p* < 0.1) are in italics. Reference: Boys of District A at T0. NI: Not included. The color scale reflects the sign and magnitude of the β coefficients: red denotes negative effects, green positive effects, and yellow coefficients close to zero.

For LSa, the higher scores among girls were largely attributable to the school-related satisfaction subscale (+8.69, *p* < 0.001) (Table 5).

**Table 5:** Life satisfaction with family, school, friends, living environment, and self scores evolution according to gender, district, and school level: Linear mixed models results.

Predictor	**Family**	**School**	**Friends**	**Living environment**	**Self**
Gender	0.28	**8**.**69**	0.05	1.04	−1.37
District^b^					
B	*−4*.*27*	−0.60	−2.53	−1.18	*−4*.*55*
C	−0.88	1.80	−0.43	3.32	**−8**.**87**
D	−2.63	0.22	−1.68	−2.16	**−4**.**90**
E	**4**.**94**	3.94	2.95	**−7**.**17**	3.18
F	−2.95	1.92	−0.32	−2.44	**−5**.**52**
Time in primary school	**0**.**70**	−0.73	**0**.**70**	0.18	0.23
Time in middle school	−0.45	**−7**.**25**	−0.54	**−3**.**21**	−1.10

Significant results (*p* < 0.05) are in bold and marginally significant results (p < 0.1) are in italics. Reference: Boys of District A at T0. The color scale reflects the sign and magnitude of the β coefficients: red denotes negative effects, green positive effects, and yellow coefficients close to zero.

In SE, overall gender differences were not significant, but girls scored higher on French SE (+5.85, *p* < 0.001) and lower on Maths SE (−2.81, *p* < 0.05) (Table 6).

**Table 6:** General, French and maths self-efficacy scores evolution according to gender, district, and school level: linear mixed models results.

Predictor	**General self efficacy**	**French self efficacy**	**Maths self efficacy**
Gender	0.90	**5**.**85**	**−2**.**81**
Girls vs Boys			
District^b^			
B	**−6**.**98**	**−6**.**85**	**−6**.**55**
C	*−4*.*31*	−0.69	0.74
D	*−3*.*33*	−2.44	−2.34
E	−1.62	0.95	−1.33
F	**−5**.**28**	−1.46	−4.12
Time in primary school	0.34	**0**.**74**	0.55
Time in middle school	−0.84	−1.82	**−5**.**97**

Significant results (*p* < 0.05) are in bold and marginally significant results (*p* < 0.1) are in italics. ^a^Boys are the reference. ^b^District A is the reference. NI: Not included. The color scale reflects the sign and magnitude of the β coefficients: red denotes negative effects, green positive effects, and yellow coefficients close to zero.

No gender-specific effects were observed in primary school. In middle school, girls showed smaller increases in LS and LSa than boys (−2.28, *p* < 0.1 for LS; −2.07, *p* ≈ 0.06 for LSa), indicating a tendency toward less pronounced improvements (Table 7).

**Table 7:** Life skills, self efficacy, life satisfaction, and health literacy scores evolution of girls compared to boys according to school level: linear mixed models results.

Outcome	Primary school (β)	Middle school (β)
Life skills	0.22	*−2.28*
Self efficacy	0.52	0.43
Life satisfaction	−0.14	*−2.07*
Health literacy	−0.36	−2.08

Significant results (*p* < 0.05) are in bold and marginally significant results (*p* < 0.1) are in italics. The color scale reflects the sign and magnitude of the β coefficients: red denotes negative effects, green positive effects, and yellow coefficients close to zero.

#### 3.3.2 District effect on life skills, self efficacy, life satisfaction and health literacy scores over time

Compared to District A, District B scored significantly lower on all scales. This difference was maintained and even reinforced after accounting for SSI, indicating socioeconomic context did not fully explain this gap. Districts D and F also showed less favorable results in SE, LSa and HL. District C scored lower in LSa and HL overall but displayed more pronounced gains in middle school, significant for LS (+5.76, *p* < 0.05) and marginal for LSa (+4.46, *p* < 0.1). District E showed slightly higher scores in LS and a small advantage in LSa (+2.46, *p* < 0.1), but this effect was strongly reduced when SSI was included (LSa: −0.45), suggesting that the apparent benefit was largely due to socioeconomic context. In terms of trajectories, District D exhibited a significantly less pronounced increase in SE during primary school (−1.65, *p* < 0.05), while District F showed smaller gains in LSa (−1.40, *p* < 0.05) and HL (−8.30, *p* < 0.05) during middle school (Table 8).

**Table 8:** Life skills, self efficacy, life satisfaction, and health literacy scores evolution of all districts according to school level: Linear mixed models results.

District^a^	School level	Life skills (*β*)	Self efficacy (β)	Life satisfaction (β)	Health literacy (β)
B	Primary	0.14	0.14	0.28	0.17
	Middle	1.33	1.63	3.85	−2.61
C	Primary	−0.03	−1.12	−0.05	0.86
	Middle	**5.76**	3.18	*4.46*	−0.06
D	Primary	*−1.07*	**−1.65**	−0.72	−1.06
	Middle	0.2	−0.97	0.3	−2.17
E	Primary	−0.24	−0.24	−0.48	0.05
	Middle	−1.57	−3.4	−1.12	−4.06
F	Primary	−0.94	−1.98	**−1.4**	−0.81
	Middle	−1.41	−5.01	*−3.64*	**−8.3**

Significant results (*p* < 0.05) are in bold and marginally significant results (*p* < 0.1) are in italics ^a^ district of reference: A. The color scale reflects the sign and magnitude of the β coefficients: red denotes negative effects, green positive effects, and yellow coefficients close to zero.

### 3.4 Sensitivity analyses

Fixed effects such as the negative association of time in middle school with LS and LSa, and the positive association of female gender with LS, remained significant and of similar magnitude in both complete-case and full datasets. However, most district effects were attenuated or reduced to statistical borderline significances, and the positive effect of female gender on SE, LSa, and HL no longer reached significance. District F remained robustly associated with lower HL in both datasets.

## 4 Discussion

### 4.1 Key findings

This quantitative longitudinal evaluation of the Explo’Santé program provides new evidence on the evolution of psychosocial and cognitive resources among pupils transitioning from primary to middle school. Three main findings stand out. First, LS, SE, and LSa increased significantly throughout primary school and into early middle school (up to T4), before experiencing a sharp decline at T5. This suggests that initial program benefits and developmental gains were not sustained during the transition to middle school. In contrast, HL followed a steadier, incremental growth over the entire period, with no collapse at T5. Secondly, gender differences were evident: girls consistently outperformed boys across all indicators. However, the gender gap narrowed at T5 due to sharper declines among girls, suggesting differential vulnerabilities during early adolescence. Thirdly, district-level disparities suggest that contextual and territorial context mediates program effects, with some districts (notably District C) showing sustained improvements, while others exhibited stagnation or even deterioration, especially during middle school.

### 4.2 Psychosocial versus health literacy trajectories

The divergent trajectories of LS, SE, and LSa compared to HL raise questions about the nature and determinants of these constructs. LS, SE, and LSa appear highly sensitive to school climate and contextual disruptions, with marked fluctuations across timepoints and an acute vulnerability during the primary-middle school transition. This pattern is supported by recent research: for example, Jarusevičiūtė et al. (2023) show that support from teachers and family significantly predict socioemotional functioning across the move to lower secondary school (45). Pedditzi et al. (46) also demonstrated that LS, SE and LSa decline more sharply around transitions, particularly among those who perceive less support (46). Longitudinal work also shows that adolescents with lower social–emotional skills and greater loneliness experience greater school burnout, implying that relational and peer climate are key predictors (47). By contrast, HL followed a steadier upward trajectory, suggesting that it depends more on cumulative cognitive exposure, structured educational input, and mediated information access. Recent studies confirm that HL scales remain stable and valid across adolescence (39), and longitudinal evidence highlights how family and educational mediation gradually strengthen HL development (48).

### 4.3 Gendered patterns

Gendered trajectories revealed both persistent advantages for girls and critical vulnerabilities. Girls scored higher in overall LS, particularly in their cognitive and social dimensions, as well as in LSa at school and SE in French. This is consistent with evidence of girls’ greater social orientation and verbal abilities (49). Boys, however, reported higher SE in mathematics, reflecting both academic gender stereotypes and differential feedback in science-related domains (50, 51). Importantly, although girls started with these advantages, their score declines at T5 were sharper, particularly in LS and LSa, suggesting a greater susceptibility to the stressors associated with the transition to middle school. This aligns with studies showing that in more gender-egalitarian contexts, girls’ satisfaction of life can decline due to heightened fear of failure or competitiveness (52). Evidence consistently shows that adolescent girls report higher levels of stress than boys, with school emerging as one of the most salient sources. Data from the HELENA study demonstrated that girls systematically perceived more stress related to school and future uncertainty than boys (53). More recently, a large mixed-method study confirmed these gender differences in school stress and identified underlying factors such as heavier school workload, emotional vulnerability, gender stereotypes, social expectations, and differential teacher treatment (54). Gendered socialization may amplify these vulnerabilities: girls may feel more pressure to maintain social bonds and academic performance, while boys may be comparatively buffered by lower socio-emotional expectations. Recent data from WHO/Europe (2024) show that school pressure has increased notably among girls, and that declines in family and peer support during adolescence are steeper for girls than for boys, suggesting that the support structures that buffer stress may erode more rapidly for girls (55).

### 4.4 Territorial and contextual inequalities

Marked differences across districts confirm that program effects are mediated by local environments. District C, despite low baseline scores, showed the most consistent gains and even resisted the T5 decline, suggesting that supportive contexts can amplify and sustain program benefits. In contrast, District E started from a favorable position but experienced the steepest decline at T5, highlighting how even seemingly advantaged settings can be vulnerable during transitions. District F, with lower SSI and weaker school climates, consistently underperformed, pointing to persistent territorial inequalities. These observations align with recent findings in France and Europe. Indeed, a scoping review showed that educational facility structures and social inequalities significantly shape children’s health and well-being outcomes, including trajectories over time (56). Similarly, the Education and Training Monitor (2024) for France documents widening socio-economic achievement gaps and stresses the importance of school climate and support in disadvantaged districts (57). In primary school settings, case study evidence shows that schools located in socially disadvantaged neighborhoods with strong local partnership and supportive leadership are better able to integrate health promotion modules effectively, suggesting that context responsiveness matters greatly for intervention success (58). These findings raise important equity concerns: without additional support, such programs risk widening existing inequalities by benefiting already favorable environments more. Addressing territorial disparities is therefore essential for ensuring the fairness and effectiveness of universal health promotion interventions.

### 4.5 The primary–middle school transition as a critical juncture

The steep decline in LS, SE, and LSa at T5 underscores the transition from primary to middle school as a period of heightened vulnerability. This transition entails not only pedagogical rupture—with greater demands for autonomy, workload, and self-regulation—but also relational discontinuity, as pupils shift from having one main teacher to multiple subject teachers, often weakening individualized socioemotional support. Evidence from recent longitudinal studies supports these patterns: for example, a German study found that LSa and school satisfaction decline statistically significantly during secondary school, particularly following the end of primary school, and that school-level achievement composition moderates these declines (59). A U. S. study of teacher-student relational warmth documents a sharp drop in perceived warmth at the transition to middle school, which predicts declines in engagement, belonging and achievement (60). In France, socio-economic gradients in academic SE become more pronounced by the end of primary school, suggesting that vulnerabilities accumulate before the transition (61). Similarly, adolescents in studies of school transition report lower school satisfaction and SE tied to the shift in environment and expectations (46). Our results suggest that the Explo’Santé program, although designed to anticipate this rupture, was not sufficient to fully buffer its disruptive effects. This finding highlights a structural challenge for school-based health promotion: while programs can strengthen psychosocial resources, broader institutional transitions and relational changes may overwhelm these gains unless systemic measures are taken.

### 4.6 Implications for health promotion programs and policies

These findings carry several implications for health promotion in schools. First, interventions should explicitly anticipate the primary-middle school transition, integrating targeted supports to maintain psychosocial gains during this critical period. Systematic reviews show that such transition-focused programs can positively affect students’ social–emotional outcomes, engagement, and self-concept when implemented around the time of transfer from primary to secondary school (62). Secondly, teacher training is crucial: educators require tools to adopt health-promoting pedagogical approaches that sustain socioemotional support alongside academic expectations. Evidence from vulnerable backgrounds indicates that a positive school climate—often maintained by teacher practices, leadership, and peer relationships—can moderate negative impacts on social and emotional skill development (63). Thirdly, territorial equity must be addressed, as program effectiveness depends heavily on local school climate and socioeconomic environments. Schools in disadvantaged contexts with poor climate or leadership tend to have less benefit from health promotion programs unless these contextual barriers are identified and directly addressed (64). Without additional resources for disadvantaged districts, universal interventions risk reproducing or widening existing inequalities (21). This underscores the relevance of the Health Promoting Schools framework, which advocates for whole-school, ecological approaches integrating curriculum, environment, and community partnerships to foster health and equity (6). More recent evidence emphasizes the importance of student engagement in HPS implementation: participatory strategies and meaningful involvement of pupils are key facilitators of success, while rigid school structures often act as barriers (65). At the same time, the Explo’Santé experience illustrates the centrality of LS in health education, but also the persistent lack of a consensual definition in research, which complicates evaluation and cross-study comparison (66). Finally, gender-sensitive approaches are needed: programs must account for differential vulnerabilities of girls and boys, tailoring strategies to support girls’ resilience during transitions and addressing boys’ persistent gaps in relational and literacy-based domains.

### 4.7 Strengths and limitations

This study has several strengths. It involved a large cohort of pupils followed longitudinally over 3 years, providing robust insights into developmental trajectories. The use of validated instruments for LS, SE, LSa, and HL ensured comparability with prior research. Multilevel modelling accounted for clustering at class and school levels, strengthening the reliability of findings. Sensitivity analyses confirmed the robustness of main effects despite attrition. However, limitations must be acknowledged. It should be noted that many scales are available to explore LS, SE, LSa and HL, with different conceptual focuses and dimensionalities. Choosing alternative measuring tools may impact the results observed. Attrition was substantial by T5, potentially introducing selection bias. Data relied on self-administered questionnaires, which may be influenced by social desirability and subjective interpretation. Importantly, no control group was available for ethical reasons, limiting causal inference on the program’s effectiveness. Moreover, the precision of district-level estimates varied substantially across the sample. Several districts included only a small number of pupils, which increases statistical uncertainty and limits the robustness of comparisons across contexts. Therefore, findings related to these smaller sub-samples should be interpreted with caution. Finally, contextual variability across districts complicates generalization, highlighting the need for replication in other educational systems.

## 5 Conclusion

This study provides new longitudinal evidence on the potential and limitations of school-based health promotion. The Explo’Santé program contributed to strengthening pupils’ psychosocial resources, confirming the relevance of embedding LS and HL into the daily functioning of schools. However, the sharp decline observed during the transition to middle school underlines the importance of reinforcing support structures at this pivotal stage of development. Gendered patterns and territorial disparities also reveal the need for context-sensitive approaches that adapt to local dynamics while ensuring equity. To better understand these differences, qualitative analyses are required, notably to identify the mechanisms, facilitators, and barriers that explain why some territories show more sustained impacts than others. Beyond its immediate outcomes, Explo’Santé illustrates how the Health Promoting Schools framework can be operationalized to address health and social inequalities from an early age, fostering environments that support both academic success and long-term well-being.

## Data Availability

The raw data supporting the conclusions of this article will be made available by the authors, without undue reservation.
