# Supplementary material for: Life skills, self-efficacy, life satisfaction and health literacy evolution from primary school to middle school: a 3-year longitudinal interventional study of the Explo’Santé cohort
Source: Front Public Health. 2026 Jan 27;14:1720265. doi: 10.3389/fpubh.2026.1720265 (PMC12886476; doi:10.3389/fpubh.2026.1720265)
Supplement: Supplementary file 1 [file Data_Sheet_1.docx]

Supplementary Material

# Supplementary Figures and Tables

For more information on Supplementary Material and for details on the different file types accepted, please see [here](https://www.frontiersin.org/guidelines/author-guidelines#supplementary-material).

## Supplementary Figures


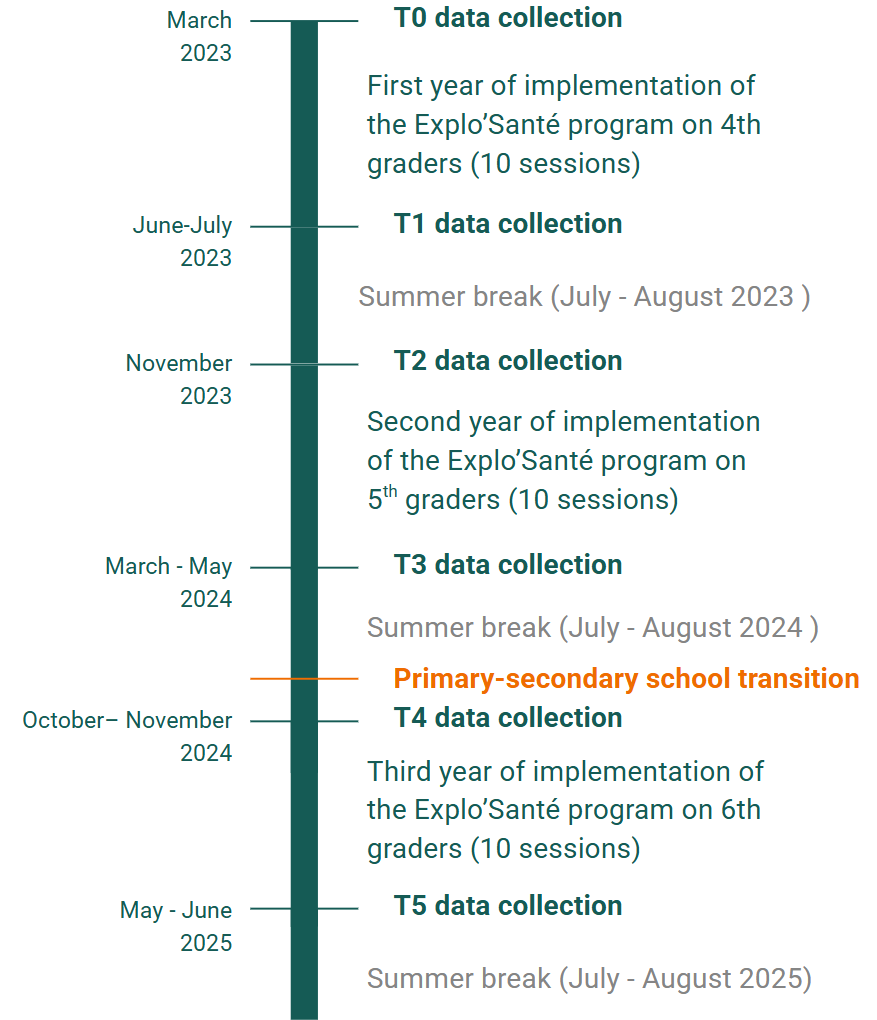


**Supplementary Figure 1** – Data collection of the Explo’Santé cohort study

## Supplementary Tables

|  | **Item No** | **Recommendation** | **Page No** |
| --- | --- | --- | --- |
| **Title and abstract** | 1 | (*a*) Indicate the study’s design with a commonly used term in the title or the abstract | 1 |
|  |  | (*b*) Provide in the abstract an informative and balanced summary of what was done and what was found | 1 |
| **Introduction** | | |  |
| Background/rationale | 2 | Explain the scientific background and rationale for the investigation being reported | 1-2 |
| Objectives | 3 | State specific objectives, including any prespecified hypotheses | 2-3 |
| **Methods** | | |  |
| Study design | 4 | Present key elements of study design early in the paper | 3 |
| Setting | 5 | Describe the setting, locations, and relevant dates, including periods of recruitment, exposure, follow-up, and data collection | 3 |
| Participants | 6 | Give the eligibility criteria, and the sources and methods of selection of participants. Describe methods of follow-up | 3 |
|  |  |  |  |
| Variables | 7 | Clearly define all outcomes, exposures, predictors, potential confounders, and effect modifiers. Give diagnostic criteria, if applicable | 4 |
| Data sources/ measurement | 8* | For each variable of interest, give sources of data and details of methods of assessment (measurement). Describe comparability of assessment methods if there is more than one group | 4 |
| Bias | 9 | Describe any efforts to address potential sources of bias | 4 |
| Study size | 10 | Explain how the study size was arrived at | 4-5 |
| Quantitative variables | 11 | Explain how quantitative variables were handled in the analyses. If applicable, describe which groupings were chosen and why | 5 |
| Statistical methods | 12 | (*a*) Describe all statistical methods, including those used to control for confounding | 5 |
|  |  | (*b*) Describe any methods used to examine subgroups and interactions | 5 |
|  |  | (*c*) Explain how missing data were addressed | 5 |
|  |  | (*d*) If applicable, explain how loss to follow-up was addressed | 5 |
|  |  | (*e*) Describe any sensitivity analyses | 5 |
| **Results** | | |  |
| Participants | 13* | (a) Report numbers of individuals at each stage of study—eg numbers potentially eligible, examined for eligibility, confirmed eligible, included in the study, completing follow-up, and analysed | 5 |
|  |  | (b) Give reasons for non-participation at each stage | 5 |
|  |  | (c) Consider use of a flow diagram | 6 |
| Descriptive data | 14* | (a) Give characteristics of study participants (eg demographic, clinical, social) and information on exposures and potential confounders | 7 |
|  |  | (b) Indicate number of participants with missing data for each variable of interest | 7 |
|  |  | (c) Summarise follow-up time (eg, average and total amount) | 7 |
| Outcome data | 15* | Report numbers of outcome events or summary measures over time | 7-11 |
| Main results | 16 | Give unadjusted estimates and, if applicable, confounder-adjusted estimates and their precision (eg, 95% confidence interval). Make clear which confounders were adjusted for and why they were included | 7-11 |
| Other analyses | 17 | Report other analyses done—eg analyses of subgroups and interactions, and sensitivity analyses | 11-15 |
| **Discussion** | | |  |
| Key results | 18 | Summarise key results with reference to study objectives | 15 |
| Limitations | 19 | Discuss limitations of the study, taking into account sources of potential bias or imprecision. Discuss both direction and magnitude of any potential bias | 18 |
| Interpretation | 20 | Give a cautious overall interpretation of results considering objectives, limitations, multiplicity of analyses, results from similar studies, and other relevant evidence | 15-17 |
| Generalisability | 21 | Discuss the generalisability (external validity) of the study results | 17-18 |
| **Other information** | | |  |
| Funding | 22 | Give the source of funding and the role of the funders for the present study and, if applicable, for the original study on which the present article is based | 19 |

*Give information separately for exposed and unexposed groups.

**Supplementary Table 1** - STROBE Statement—Checklist of items that should be included in reports of cohort studies

| **District** | **Boys N (%)** | **Girls N (%)** | **Primary schools socioeconomic status index^a^** | **Middle schools socioeconomic status index^a^** |
| --- | --- | --- | --- | --- |
| A | 504 (45.2%) | 612 (54.8%) | 108.6 (3.7) | 110.1 (0.0) |
| B | 204 (50.0%) | 204 (50.0%) | 103.5 (2.1) | 102.4 (0.0) |
| C | 192 (48.5%) | 204 (51.5%) | 102.4 (10.4) | 101.3 (0.5) |
| D | 516 (48.1%) | 558 (51.9%) | 96.1 (2.1) | 93.1 (0.0) |
| E | 444 (49.3%) | 456 (50.7%) | 85.3 (7.2) | 78.6 (2.2) |
| F | 264 (46.3%) | 306 (53.7%) | 95.3 (6.5) | 92.1 (0.0) |

**Supplementary Table 2.** Gender distribution and mean socioeconomic index (SSI) of each district. ^a^Mean, standard deviation.

| **Gender** | **Time** | **Life skills^a^** | **Self efficacy^a^** | **Life satisfaction^a^** | **Health literacy^a^** | **N** |
| --- | --- | --- | --- | --- | --- | --- |
| **All** | **T0** | 60.5 (14.6) | 70.6 (17.0) | 74.9 (12.7) | 69.0 (17.6) | 662 |
|  | **T1** | 60.2 (14.6) | 71.8 (17.3) | 74.9 (13.3) | 71.6 (16.6) | 659 |
|  | **T2** | 61.1 (14.2) | 72.9 (17.2) | 77.0 (13.6) | 70.5 (17.9) | 705 |
|  | **T3** | 61.5 (14.9) | 73.1 (17.0) | 76.2 (13.9) | 71.5 (17.9) | 675 |
|  | **T4** | 63.6 (13.4) | 74.8 (15.1) | 77.2 (12.6) | 72.2 (17.7) | 472 |
|  | **T5** | 60.4 (15.5) | 70.8 (18.5) | 74.3 (14.3) | 73.1 (18.6) | 443 |
| **Boys** | **T0** | 58.8 (15.4) | 70.2 (17.8) | 73.7 (13.4) | 67.5 (18.7) | 354 |
|  | **T1** | 58.2 (14.8) | 71.0 (18.3) | 73.1 (13.5) | 70.7 (16.8) | 354 |
|  | **T2** | 58.9 (14.5) | 71.9 (18.5) | 75.9 (13.8) | 69.6 (19.1) | 354 |
|  | **T3** | 59.2 (15.7) | 72.0 (17.8) | 75.7 (13.7) | 70.1 (18.4) | 354 |
|  | **T4** | 61.5 (14.2) | 74.5 (15.9) | 76.4 (12.8) | 70.5 (18.3) | 354 |
|  | **T5** | 59.7 (16.7) | 70.4 (19.8) | 73.9 (14.4) | 72.9 (20.0) | 354 |
| **Girls** | **T0** | 62.1 (13.6) | 71.0 (16.3) | 75.9 (12.0) | 70.4 (16.4) | 390 |
|  | **T1** | 62.1 (14.2) | 72.5 (16.3) | 76.5 (13.0) | 72.4 (16.4) | 390 |
|  | **T2** | 63.0 (13.7) | 73.8 (15.8) | 78.0 (13.4) | 71.3 (16.7) | 390 |
|  | **T3** | 63.6 (14.0) | 74.0 (16.3) | 76.6 (14.0) | 72.8 (17.5) | 390 |
|  | **T4** | 65.4 (12.5) | 75.0 (14.5) | 77.8 (12.5) | 73.7 (17.1) | 390 |
|  | **T5** | 60.9 (14.3) | 71.2 (17.3) | 74.6 (14.3) | 73.2 (17.2) | 390 |

**Supplementary Table 3.** Life Skills, Self Efficacy, Life Satisfaction, and Health Literacy mean scores for all students and according to gender at each time point. ^a^Mean (standard deviation).

| **District** | **Time** | **Life skills** | **Self efficacy** | **Life satisfaction** | **Health literacy** | **N** |
| --- | --- | --- | --- | --- | --- | --- |
| **A** | T0 | 61.4 (14.2) | 72.5 (16.0) | 75.1 (11.7) | 71.5 (16.1) | 186 |
|  | T1 | 60.9 (14.0) | 73.6 (17.0) | 74.6 (12.6) | 74.2 (15.6) | 186 |
|  | T2 | 63.1 (13.2) | 76.9 (16.0) | 78.8 (12.1) | 74.7 (15.3) | 186 |
|  | T3 | 62.9 (12.8) | 75.5 (16.7) | 77.1 (12.7) | 73.8 (17.6) | 186 |
|  | T4 | 63.4 (13.7) | 76.0 (15.1) | 77.8 (11.9) | 73.4 (17.6) | 186 |
|  | T5 | 60.4 (15.6) | 73.2 (18.0) | 74.1 (13.4) | 77.6 (20.8) | 186 |
| **B** | T0 | 56.1 (14.4) | 63.5 (18.3) | 71.4 (13.5) | 66.2 (18.3) | 68 |
|  | T1 | 58.8 (15.2) | 67.5 (17.6) | 76.1 (13.3) | 70.8 (19.9) | 68 |
|  | T2 | 57.1 (14.4) | 68.9 (16.9) | 74.5 (13.2) | 68.6 (18.3) | 68 |
|  | T3 | 59.1 (15.2) | 70.7 (16.6) | 75.7 (14.1) | 69.2 (17.7) | 68 |
|  | T4 | 60.1 (12.6) | 70.9 (16.9) | 73.3 (12.7) | 67.8 (17.1) | 68 |
|  | T5 | 58.9 (14.4) | 69.0 (16.0) | 75.5 (11.4) | 70.9 (18.1) | 68 |
| **C** | T0 | 57.9 (13.2) | 70.6 (13.6) | 73.5 (13.2) | 63.4 (18.8) | 66 |
|  | T1 | 60.5 (14.8) | 71.6 (16.7) | 74.1 (12.6) | 70.4 (16.7) | 66 |
|  | T2 | 61.1 (13.6) | 73.2 (16.0) | 76.4 (14.2) | 67.7 (19.9) | 66 |
|  | T3 | 59.4 (14.7) | 72.9 (16.9) | 74.0 (14.9) | 69.6 (16.1) | 66 |
|  | T4 | 63.9 (12.7) | 75.8 (13.3) | 76.6 (13.2) | 72.4 (18.4) | 66 |
|  | T5 | 65.6 (12.8) | 75.8 (17.3) | 77.7 (15.3) | 74.5 (14.6) | 66 |
| **D** | T0 | 60.6 (16.2) | 70.6 (17.5) | 73.6 (13.4) | 69.8 (19.2) | 179 |
|  | T1 | 60.0 (16.0) | 72.6 (17.4) | 73.7 (14.1) | 71.6 (17.5) | 179 |
|  | T2 | 60.9 (15.8) | 70.7 (17.4) | 75.4 (15.4) | 69.1 (20.4) | 179 |
|  | T3 | 60.4 (16.2) | 71.2 (17.3) | 75.0 (15.1) | 70.2 (20.1) | 179 |
|  | T4 | 64.0 (15.7) | 73.1 (15.4) | 76.3 (14.2) | 69.8 (18.7) | 179 |
|  | T5 | 61.2 (16.7) | 70.7 (18.9) | 74.1 (15.9) | 72.4 (18.0) | 179 |
| **E** | T0 | 63.3 (12.9) | 72.9 (16.8) | 78.7 (11.1) | 70.7 (16.6) | 150 |
|  | T1 | 60.4 (14.0) | 71.3 (17.3) | 76.5 (14.6) | 70.7 (14.7) | 150 |
|  | T2 | 61.7 (13.4) | 75.4 (15.9) | 80.2 (13.7) | 71.7 (16.4) | 150 |
|  | T3 | 64.5 (13.8) | 76.0 (16.9) | 79.8 (13.1) | 74.7 (15.9) | 150 |
|  | T4 | 65.1 (11.5) | 76.6 (13.5) | 80.6 (11.0) | 76.5 (16.3) | 150 |
|  | T5 | 58.3 (15.8) | 68.3 (19.9) | 74.8 (14.7) | 73.2 (17.8) | 150 |
| **F** | T0 | 59.6 (14.6) | 69.4 (17.7) | 74.7 (13.4) | 66.4 (15.9) | 95 |
|  | T1 | 59.9 (13.2) | 70.2 (17.5) | 74.9 (11.5) | 69.3 (16.9) | 95 |
|  | T2 | 59.5 (14.0) | 68.7 (19.2) | 74.5 (11.8) | 66.4 (16.5) | 95 |
|  | T3 | 59.7 (17.2) | 69.1 (16.6) | 72.8 (13.3) | 68.0 (18.5) | 95 |
|  | T4 | 64.2 (13.3) | 74.2 (16.8) | 76.0 (13.1) | 69.9 (17.4) | 95 |
|  | T5 | 59.2 (15.1) | 67.0 (18.8) | 70.8 (14.5) | 64.8 (15.6) | 95 |

**Supplementary Table 4**. Life Skills, Self Efficacy, Life Satisfaction, and Health Literacy mean scores for each district at each time point. ^a^Mean (standard deviation).

| **Self efficacy survey (6-item Likert scale)** |
| --- |
| **Self efficacy at school** |
| J’ai des bonnes notes à l’école. |
| Le travail à l’école n’est pas très difficile. |
| Comme je suis bon(ne) à l’école, je peux résoudre tous les exercices que l’on me pose. |
| Lorsque j’ai un problème dans un exercice, je me débrouille toujours pour trouver la solution. |
| Même si c’est très difficile, j’essaie plusieurs fois d’y arriver. |
| Si l’exercice est très difficile, je cherche un moyen pour réussir quand même à trouver la solution. |
| **Self efficacy in French** |
| J’arrive toujours à finir mes exercices de français. |
| J’arrive à me concentrer sur mes exercices de français à l’école. |
| Je comprends les exercices de français. |
| J’arrive à me motiver pour faire mes exercices de français en classe. |
| Je suis capable de m’organiser pour faire mes exercices de français en classe. |
| Je suis capable d’écrire les leçons de français tout seul. |
| **Self efficacy in Maths** |
| J’arrive toujours à finir mes exercices de maths. |
| J’arrive à me concentrer sur mes exercices de maths à l’école. |
| Je comprends les exercices de mon fichier de maths. |
| J’arrive à me motiver pour faire mes exercices de maths. |
| Je suis capable de m’organiser pour faire mes exercices de maths en classe. |

| **Life satisfaction survey (4-item Likert scale)** |
| --- |
| **Life satisfaction with family** |
| J'adore être à la maison avec ma famille. |
| J'adore passer un peu de temps avec mes parents. |
| Ma famille est sympa. |
| On s'entend bien dans ma famille. |
| Mes parents me traitent équitablement. |
| Les membres de ma famille parlent cordialement les uns avec les autres. |
| J'ai la meilleure famille qui soit. |
| Je passe des moments sympas avec mes parents. |
| **Life satisfaction with school** |
| J'aime bien être à l'école. |
| Je suis impatient d'aller à l'école. |
| L'école est quelque chose d'intéressant. |
| J'aimerais ne pas aller à l'école. |
| J'adore les activités scolaires. |
| J'apprends plein de choses à l'école. |
| Il y a beaucoup de choses que je n'aime pas à l'école. |
| **Life satisfaction with friends** |
| Mes amis sont sympas avec moi. |
| Mes amis sont gentils avec moi. |
| Mes amis sont excellents. |
| Mes amis m'aident si j'ai besoin d'eux. |
| Je passe du bon temps avec mes amis. |
| **Life satisfaction with home** |
| J'aime bien habiter ici. |
| J'adore le quartier où j'habite. |
| J'aimerais habiter dans une maison différente. |
| J'aimerais habiter ailleurs. |

| **Life satisfaction with self** |
| --- |
| Je pense que je suis belle/beau. |
| Je suis quelqu'un avec qui on s'amuse. |
| Je m'aime bien. |
| La plupart des gens m'apprécient. |
| Je sais faire pas mal de choses. |
| Je suis quelqu'un de bien. |

| **Life skills survey (5-item Likert scale)** |
| --- |
| **Cognitive life skills** |
| Pour moi c’est facile de me fixer des buts pour l’apprentissage en classe. |
| J’organise bien mon travail en classe. |
| Quand je lis un problème, je m’assure de bien comprendre ce qui est demandé avant de commencer. |
| Avant de commencer un exercice/problème, j’essaie de me le représenter dans ma tête. |
| Quand j’ai fini de faire un problème/exercice, je vérifie si je n’ai pas fait d’erreurs. |
| Quand je révise pour un contrôle, je relis mon cahier et refais les exercices. |
| Quand je révise pour un contrôle, j’utilise les exercices corrigés dans mes cours ou dans le livre afin de comprendre les différentes étapes de résolution. |
| Je trouve que travailler sur des exercices déjà résolus est une bonne manière de réviser. |
| Quand je n’arrive pas à faire les exercices du livre, je regarde la solution à la fin du livre si elle est disponible. |
| Quand je n’arrive pas à résoudre un problème, j’essaie de trouver quelqu'un qui pourra le faire pour moi. |
| Quand je lis quelque chose que je ne comprends pas, je le passe en espérant que la maitresse ou le maitre l’expliquera en classe. |
| Quand dans mes devoirs il y a un problème difficile à résoudre, je renonce et passe au problème suivant. |
| **Emotional life skills** |
| J’arrive à me calmer après avoir reçu une mauvaise note. |
| Quand je reçois une mauvaise note, je prends une grande respiration pour ne pas me mettre en colère. |
| Je prends une grande respiration pour me calmer après avoir reçu une mauvaise note. |
| Si je n’arrive pas à faire mes devoirs, je reste quand même positif. |
| J’ai une attitude positive lorsque je suis face à une difficulté à l’école. |
| À l'école, je pense positivement même si j'ai des difficultés pour apprendre. |

| **Social life skills** |
| --- |
| Je sais résoudre calmement les désaccords qui m’opposent à mes amis. |
| Quand j’ai un conflit avec un camarade de classe, je le résous calmement par la discussion. |
| Quand j’ai un conflit avec un camarade de classe, je recherche une solution qui puisse nous satisfaire tous les deux. |
| Si je me dispute avec un camarade, j’essaie de résoudre le problème par la discussion. |
| Quand mes amis ont un problème, je leur donne de bons conseils. |
| Je sais aller vers les autres quand ils ont un problème. |
| Je réconforte mes amis quand ils ne vont pas bien. |
| J’aide les autres en écoutant leurs problèmes. |
| Quand j'ai un problème, je trouve des personnes qui me montrent le bon exemple pour m'aider. |
| Quand j'ai un problème, je cherche des personnes qui savent m’écouter et m'aider. |
| Quand j'ai un problème, je cherche de l'aide auprès des personnes qui m'encouragent. |
| Quand j'ai un problème, je vais vers les gens qui vont m'aider à le résoudre. |

| **Health literacy survey (4-item Likert scale)** |  |
| --- | --- |
| **Items** |  |
| Je pense que je suis bien informé.e sur la santé. |  |
| Je pense que si besoin, je suis capable de donner des idées pour améliorer la santé autour de moi (par ex : amis, famille, voisins, quartier) |  |
| Je pense que je sais comparer des informations sur la santé qui viennent de différentes sources (par ex : internet, presse, amis). |  |
| Je pense que je suis capable de suivre les consignes que me donne le personnel soignant (par ex infirmière, médecin). |  |
| Je pense que je peux facilement donner des exemples des choses qui sont bonnes ou mauvaises pour la santé. |  |
| Je pense que je suis capable d’évaluer en quoi mes actions ont un effet sur la nature autour de moi. |  |
| Je pense que si besoin, je trouve des informations sur la santé que je comprends facilement. |  |
| Je pense que je suis capable d’évaluer en quoi mon comportement à un effet sur ma santé. |  |
| Je pense que, en général, j’arrive à déterminer si une information sur la santé est vraie ou fausse. |  |
| Je pense que je peux donner les raisons des choix que je fais concernant ma santé. |  |

**Supplementary Table 5.** Life Skills, Self Efficacy, Life Satisfaction, and Health Literacy scales used in the Explo’Santé study and their items.
